# Construction of knowledge and perception of mammography in the UK

**DOI:** 10.3332/ecancer.2008.98

**Published:** 2008-09-30

**Authors:** M Takechi

**Affiliations:** School of Social Sciences, Cardiff University, Cardiff, UK

## Abstract

**Objective:**

The benefit of mammography is overestimated among UK women, although the National Health Service (NHS) offers comprehensive information about breast screening with its official pamphlet. This study examined how women in the UK construct their views of mammography and how they interpret the information in the NHS pamphlets and newspaper articles about breast screening.

**Methods:**

Focus groups and individual interviews with 11 female participants aged 26–58 were conducted using a baseline questionnaire.

**Results:**

Many participants possessed knowledge about mammography, which differed from the medical consensus. Various factors, including self-beliefs, experience of breast cancer or being screened with a mammogram and stories from friends and relatives, could influence the participant’s view about breast screening with a mammogram. Sceptical attitudes towards the media description of breast screening issues were revealed. The participants felt that the NHS pamphlets offered enough information about breast screening.

**Conclusion:**

The women in this study showed that people may not be surrounded by the practical information sources to know about mammogram efficacy until they are invited to the breast screening. In order to achieve democratic discussion over breast screening, including mammography, the NHS and mass media have room for further cooperation to provide the full picture of breast screening to the public.

## Introduction

The NHS Breast Screening Programme (NHS BSP), which invites women aged between 50 and 70 to breast screening three yearly, has improved its breast cancer detection rate since foundation [1]. It has been suggested that public members should be given conclusive information about health screenings [2,3].

Many countries where a breast screening programme is organized offer official pamphlets covering a range of information about mammography [4]. Nevertheless, research has shown that the public tends to overestimate the efficacy of mammography [5,6]. A quantitative study by Domenighetti *et al* [7] shows that the tendency to overestimate the efficacy of mammography among UK women was higher than that of women in the US and Switzerland, where there were no governmental breast screening programmes [7]. This is possibly due to the appropriation of official information about breast screening caused by trying to achieve the governmental aim as to high-screening rate [8].

The 2003 report of the House of Lords Select Committee on Science and Technology, while admitting the importance of the media role in communicating science to the public, noted scientists’ dissatisfaction with media portrayal of scientific issues [9]. One recent study showed that news media tend to lead the public to understanding only major topics regarding scientific issues, not the research facts or scientific outcome, which back up those topics [10].

The UK Chief Medical Officer (CMO) stresses that members of the public are encouraged to be committed to the debate of health care issues rather than just consume what the authorities have decided [11]. A qualitative study focusing on US women revealed that, on constructing the perception of mammography, the participants had many influential factors such as psychological characteristics and the information from medical experts, personal experiences and interpersonal communication [12].

The purpose of this study was to explore how members of the public in the UK, especially in the Cardiff area, construct their views of mammography, in order to address whether they are surrounded by practical information sources. Furthermore, this study sought to look in to how the participants perceive the information about mammography from both the NHS and the media, as they are important information bases for public engagement in the breast screening debate.

## Methods

This study used a qualitative research method in order to explore how the participants had constructed their perception of mammography. A focus group method was adopted as the main strategy as this method allows participants to feel more comfortable and express their real perspectives [13]. Two groups were prepared: a group of women who had been screened with a mammogram and a group of women who had not. An individual interview measure was also prepared for those who could not participate in focus groups. This research was given ethical approval from the School Research Ethics Committee of Cardiff School of Social Sciences, and participants were given the fully informed written consent of the study.

Women over the age of 20 were recruited in the Cardiff area by distributing handouts at some shops and cancer charity offices as well as by using the electronic notice board system of Cardiff University computer network. Seventeen women were contacted through the University’s notice board, and 11 women aged between 26 and 58 actually participated in either focus groups or individual interviews at Cardiff University during November and December 2007. For women who had not been screened with a mammogram, one focus group with three participants and three individual interviews were carried out. Another focus group and three individual interviews were conducted for women who had been screened with a mammogram before.

There was one participant who had a family history of breast cancer in each group. Three women who did not have a mammogram experience, and one woman who had a mammogram experience, had friends affected by breast cancer. There were two participants who had had breast cancer in the group of women with a mammogram experience. One participant had not been screened with a mammogram although her age was over 50.

At the beginning of the focus group or individual interview, participants were asked to fill out a baseline questionnaire. This questionnaire aimed to assess participants’ basic awareness of breast screening with a mammogram. Table 2 is a summary of the questionnaire. Three questions, from Question 3 to 5 were taken from the study of Domenighetti *et al* [7].

The actual interviews started by asking the following introductory questions: (1) What do you think about breast cancer? (2) What do you consider as risk factors of contracting breast cancer? and (3) What is mammography to you? After these introductory questions, the participants were asked about: (4) influential factors or information sources in constructing their views of mammography or in deciding to take a mammogram. On interviewing the participants, the influential factors or information sources were divided into the following four categories: (a) media coverage, (b) friends and relatives, (c) medical experts and (d) other factors or information sources. The participants were also asked to give the information content they obtained from those sources.

In the last part of each session, the participants were asked to express their views on reading the information on mammography from the following two sources: (5) the NHS BSP pamphlets, and (6) two types of newspaper coverage. One of the pamphlets was named ‘Breast Screening: A Pocket Guide’, and the other was entitled ‘Breast Screening: The Facts’ [15], which was subsidiary to the first one. The first newspaper coverage was headlined ‘The breast scans may cause cancer in high-risk women’ from the *Daily Mail* [16]. The second one was from the *Daily Telegraph* and contained an article headlined ‘Breast screening for under-50s questioned’ [17] and one radiologist’s response to this article titled ‘Early detection may have saved my life’ [18].

Each conversation from focus groups and individual interviews was transcribed and analysed carefully along with the questionnaire results and the field notes created through the sessions.

## Results

### A. Questionnaire results: perspectives on the efficacy of mammography

For Question 1, asking ‘Who do you think the NHS Breast Screening Programme invites to the screening?’, all the participants answered that women over the age of 50 are invited to the NHS BSP.

Question 2—‘Why do you think the NHS set the standard as you stated?’—saw eight participants suggest that women aver the age of 50 were most at risk of contracting breast cancer so the NHS invited this age group. Two women with a mammogram experience answered that cost-effectiveness was also attributable to the age criteria that NHS had set. One participant did not know the reason.

Answers to Questions 3–6 are summarized in Table 3. Only one participant had all correct answers through Questions 3–5, while the other ten participants tended to either answer ‘don’t know’ or overestimate.

For the outcome of Question 3, four women without a mammogram experience answered ‘don’t know’. In the group of women with a mammogram experience, two participants chose the answer ‘don’t know’ and the other three participants selected overestimated answers.

With regard to Question 4, three women without a mammogram experience answered ‘don’t know’, and two women in this group selected the correct answer. Four women with a mammogram experience picked overestimated answers.

As to Question 5, no participants chose the answer ‘don’t know’. Three participants of each group gave overestimated answers. Three women without a mammogram experience and two women with a mammogram experience selected the correct answer.

For the answers to Question 6, five participants mentioned self-examination as an important breast examination method. Three participants answered that they did not know whether mammography is most effective. One participant confided that mammography was the most effective breast screening method.

## B. Interview results

### (1) The concept of breast cancer

Five participants conceptualized breast cancer as a terrifying disease. Three participants answered that if tumours are caught early enough, there may be various treatments available. The other three participants were not worried about contracting breast cancer.

### (2) Risk factors of contracting breast cancer

All the participants gave genetics as a risk factor of contracting breast cancer. Diet was also suggested by six participants. Being post-menopausal was given by two women with a mammogram experience. Four women associated smoking as one risk factor of contracting breast cancer.

### (3) Interpretation of mammography

In a group of women who had not been screened with a mammogram, four participants insisted how uncomfortable a mammogram procedure would be. Of a group of women who had been screened with a mammogram, three participants expressed that the benefit of mammography outweighed the discomfort of the procedure.

### (4) Factors influencing participants’ view of mammography

#### (a) Media coverage

No participants could recall any specific media coverage of mammography. Two women without a mammogram experience had seen the coverage about the NHS BSP, but the report was not specifically about mammography. One woman stated that she possibly had been influenced by the media coverage from ‘Let’s go for the screening’ point of view. Four women expressed doubt as to whether the mass media could be a reliable source for information about mammography.

‘I would get an idea, but I would look at it further, I wouldn’t take it as a face value’. (Participant no. 8)

‘(Media coverage) is a short term thing’. (Participant no. 11)

#### (b) Friends and relatives

Six women possessed experience of having conversations about mammography with their friends or relatives. Four of them without a mammogram experience stated that the conversation was about the feeling of those friends or relatives on taking their mammogram. Another participant stated that her friend whose tumour was found by a mammogram, had strongly influenced on her view that mammography is very important. One woman over the age of 50, who had not been to the NHS BSP before, denied her friend’s influence over her, as she herself believed that the mammogram procedure is not friendly to women.

‘I’m assured when I speak to someone who agrees with me, but most people don’t agree with me, so I could hardly be influenced’. (Participant no. 4)

#### (c) Medical experts

There was only one participant who had an experience of having conversation about mammography with her general practitioner (GP) before being invited or having any breast problem. Three women took a mammogram because their symptoms were found by their GPs. In addition, two participants implied people’s dependence on GPs or medical experts to find out abnormalities in their bodies. Also, two women stated that they would visit their GPs if they had any concern about breast cancer.

#### (d) Other sources or influential factors

As to other useful sources of information about mammography, four women suggested the internet as available in their daily lives, and three participants considered medical journals as practical. The other three participants regarded cancer charities as useful since those charities offered members of the public the information about breast cancer or detecting methods in a more familiar way. Two women with a mammogram experience stated that they had referred to pamphlets of the NHS BSP on taking their mammograms.

During the interviews, many participants revealed that they had not considered mammography until the time of this study’s sessions, an invitation to the NHS BSP, or an encounter with breast cancer.

‘I wouldn’t have thought anything about it (mammography) at all until the letter came inviting me for the screening’. (Participant no. 8)

### (5) Interpretation of information from the NHS pamphlets

Participants revealed their own impression on reading two types of NHS pamphlets. Most participants admitted that the NHS pamphlets offered enough information. They also suggested that there was an appropriate amount of figures in the pamphlets.

‘It’s not patronising. It’s not offensive. It’s not frightening. It’s just all the facts. It’s clearly explained’. (Participant no. 2)

‘It is informative. There’s not a lot in there that it’s missed’. (Participant no.9)

Some participants appreciated that the NHS offered a subsidiary booklet explaining about the NHS BSP without scientific figures.

‘That (the Facts) is very good one for someone who isn’t gonna read a lot of stuff’. (Participant no.11)

However, one woman without a mammogram experience considered that the pamphlets did not cover enough information about the method of mammography itself. In addition, some women gave comments that the pamphlet portrayal of the information could be more reassuring and less confusing.

‘Lots of statistics. I’d like to see a pie chart rather than all these figures’. (Participant no. 4)

‘Ninety-five per cent as normal of those we (the NHS) called. It would be better if they said 5% or the numbers about these abnormalities. Don’t fudge it up’. (Participant no. 8)

### (6) Interpretation of the information from newspaper articles

Reading an article from the *Daily Mail*, seven participants showed their confusion or dissatisfaction regarding what was described in the article.

‘Scaremongering. They can’t get the facts right from one single article’. (Participant no. 1)

‘I’ve got the impression that mammography is bad’. (Participant no. 5)

‘I really don’t know what to think about’. (Participant no. 6)

Two women without a mammogram experience stated that people would consider the words ‘breast scans’ of the title as ‘mammograms’.

‘I tend to flick through papers. If I read “breast scans may cause cancer in high-risk women”, I would equate that mammograms cause cancer in high-risk women’. (Participant no. 3)

The *Daily Telegraph* coverage also left the participants with some confusion and dissatisfaction. Especially, one sentence: ‘Even though screening at a younger age caused 17% drop in breast cancer mortality, the result was not statistically significant’ seemed to catch the attention of many participants.

‘I would have to read the Lancet or whatever to get the real picture’. (Participant no. 4)

‘Who wrote this? Who is questioning the significant 17% drop in mortality?’ (Participant no. 6)

Most participants showed a sceptical attitude about news media itself.

‘The papers, they always take a negative view and blow it up’. (Participant no. 4)

‘I certainly don’t believe everything I read in the newspaper’. (Participant no. 9)

## Discussion

It has been suggested that women tend to overvalue how effective mammography would be [5,6,12]. By conducting the baseline questionnaire, it was revealed that the majority of women in my study had an overestimated view of the efficacy of mammography. When asked two questions regarding the proportion of lives saved and the reduction in the rate of mortality achieved with mammogram screening, women without mammogram experience tended to choose the answer ‘don’t know’; meanwhile, women with a mammogram experience never selected the answer ‘don’t know’, but were likely to select overestimated answers. These questionnaire results might suggest that it was difficult for women to know about the efficacy of mammography unless they had been screened with a mammogram. Furthermore, even if women experience being screened with a mammogram, it did not necessarily lead them to possess a correct understanding of mammogram efficacy. Further research is recommended on whether cancer fear motivates people to seek the appropriate information about breast screening methods.

Over half of participants accepted the idea that mammography could prevent or reduce the risk of contracting breast cancer, despite the fact that mammography could be only a method of breast cancer screening. In addition, most participants persisted that self-awareness is significant as a method of breast screening. It is ironic that although they were aware of the importance of self-awareness to screen breast cancer, they did not understand the true sense of mammography as a breast cancer screening method.

The interview sessions explored what kind of factors had influenced the participant’s view of mammography. The participants remembered media coverage of breast screening or conversations about mammography with their friends, but neither information sources were practical for the participants to know about mammogram efficacy. Women with a mammogram experience tended to have had an interaction with their GP in the process of finding their breast problems. Nevertheless, these interactions happened in order only to make a referral for screening with a mammogram so that those participants never talked about the efficacy of mammography with their GP at that time.

Members of the public basically possess high trust in health authorities to have the best knowledge of medicine [19]. Many participants did indeed admit that GPs or medical experts would be reliable when breast problems occurred. As a whole, it was suggested that participants would basically rely on medical experts concerning breast cancer; however, for the participants, visiting GPs did not necessarily mean obtaining information about the efficacy of mammography.

Only women being invited to the NHS BSP had had an opportunity to read the official pamphlet about breast screening. Furthermore, some participants stated that mammography was not an issue they would consider unless they were faced with a particular occasion, such as having breast problems or being invited to the NHS BSP. It might be suggested that women in this study were not surrounded by enough opportunities, unless they had been invited to the breast screening, to know about the efficacy of mammography.

Achieving a clear understanding of breast screening among the general public is a common issue faced by all health organizations [4]. Most women in this study suggested that the official pamphlet provides enough information about mammography. On the other hand, some participants mentioned that the explanation of figures in the pamphlets was confusing or too reassuring. In order to achieve a clearer understanding of breast screening among the general public, it may be necessary for the NHS BSP to improve the way statistics are described in the official pamphlet.

Mass media, when dealing with health issues, can exaggerate risk in order to make profit [20]. In fact, this study revealed the participants’ dissatisfaction with the news media since the paper articles described breast screening issues in an incomprehensive way. Moreover, people are likely to remember only the main topic from seeing media coverage of scientific issues, but not to recall the scientific facts related to that topic [10]. Some participants in this study did indeed understand only the idea put forward by the news articles and not what the original research had revealed. This may be partly because the newspaper articles manipulated the information about the original research in a complicated way. News media often bring new aspects regarding health issues [21]. The news articles used in my study also dealt with new scientific medical research. However, because the articles’ descriptions were not acceptable, the participants did not consume those new findings as a realistic value. To avoid this unfortunate situation, news media should gain credit amongst the public by seeking a more lucid way of explaining research facts.

In this study, both the results from the questionnaire and interviews may indicate that there was not enough information provided for the participants to understand the efficacy of mammography. Considering the CMO’s suggestion that the public should be committed to health care debates [11], the participants seemed not to be fully ready to be involved in the breast screening debate. In order to pursue the public involvement in the breast screening debate, it would be necessary to provide a full picture of mammography. Moreover, that full picture should be delivered to society in the way that the public can understand easily and clearly. Responsibility for giving conclusive information about breast screening is attributable to the NHS, considering the high public trust in health authorities. Nevertheless, mass media should also be encouraged to develop their coverage of breast screening stories as a supportive means for health or science organizations to communicate with the public [9] and as the representative of the public voice about health issues [22]. In this way, we could achieve not only informed choice of health care among public members but also public involvement in health care debates.

In this study, there were two participants who had a strong personal opinion about either mammography itself or her taking a mammogram. In these cases, those two women tended to have been persistent in their own opinions over any information about mammography. Further research could explore the influence of those personal beliefs on participants’ attitudes towards obtaining the information about mammogram efficacy. Therefore, it would be able to suggest how and to what extent health authorities should intervene with those people to increase their understanding about the efficacy of mammography.

In this study, it was not possible to assess whether holding a science degree led to the participants’ appropriate knowledge about the efficacy of mammography. Furthermore, 11 participants had limited demographic and sociodemographic backgrounds, including the fact that they were from Cardiff University, an academic institution. Quantitative research with participants from various demographic and/or sociodemographic backgrounds may offer different outcomes or further support to generalize this study result.

## Figures and Tables

**Table 1:**
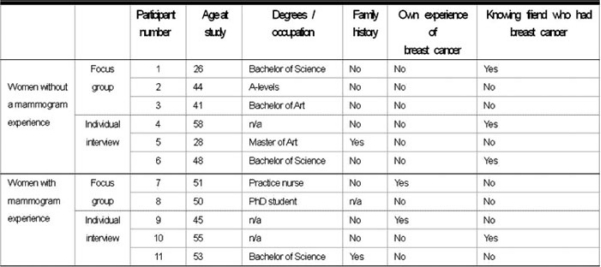
Participants’ demographic characteristics

**Table 2:**
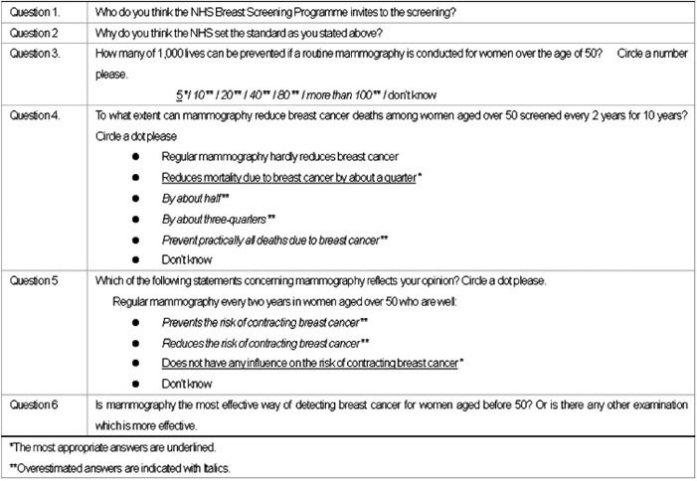
Question from baseline questionnaire

**Table 3:**
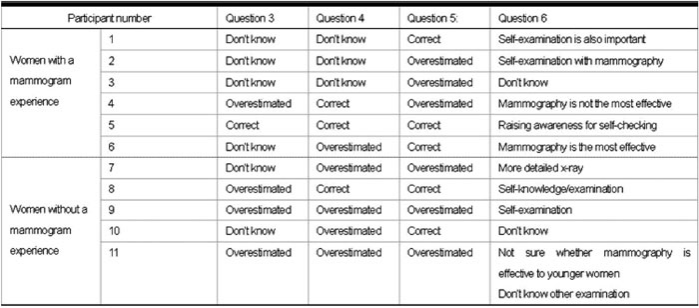
Participants’ perspectives regarding the efficacy of mammography
